# Electronic Spectroscopy of Cold Gas‐Phase Ions in a Cryogenic Ion Trap: Vibronic States, Bonding Characteristics, and Photochemistry

**DOI:** 10.1002/asia.70633

**Published:** 2026-02-23

**Authors:** Satoru Muramatsu, Masahiro Koyama, Yoshiya Inokuchi

**Affiliations:** ^1^ Department of Chemistry, Graduate School of Advanced Science and Engineering Hiroshima University Higashi‐Hiroshima Japan

**Keywords:** cryogenic ion trap, electronic spectroscopy, gas‐phase ions, photochemistry, vibronic states

## Abstract

Laser spectroscopy under cryogenic gas‐phase conditions enables the high‐precision investigation of intrinsic molecular properties by minimizing perturbations from external environments such as impurities, solvents, and counterions. In particular, cryogenic ion‐trap (CIT) spectroscopy has broadened access to a diverse range of molecular and cluster ions. When combined with electronic ultraviolet–visible (UV–Vis) spectroscopy, which is typically performed in action schemes (photofragmentation, evaporation of inert tag molecules, laser‐induced fluorescence, and so on), it serves as a sensitive and accurate probe for the vibronic structures, bonding characteristics, conformations, and excited‐state dynamics of target ions. In this review, we present a brief overview of typical experimental setups and key techniques for CIT spectroscopy; we then survey recent case studies for various ions, including carbocations and protonated hydrocarbons, organic dyes, host–guest complexes, microhydrated ions, hypervalent ions, metal clusters, and chemical intermediates formed in solution. We highlight the precise and unambiguous information accessible using this method and illustrate the rapidly expanding scope of modern gas‐phase chemistry.

## Graphical abstract

Recent development of cryogenic ion‐trap‐based instrumentation has extended the scope of gas‐phase laser spectroscopy to a wide variety of nonvolatile ions, including carbocations and protonated hydrocarbons, organic dyes, host‐guest complexes, micro‐hydrated ions, hypervalent ions, metal clusters, and ionic intermediates formed in solution.

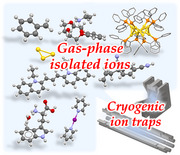

## 1 Introduction

### 1.1 Cryogenic Ion‐Trap Spectroscopy

Isolating molecules and ions in the gas‐phase yields chemical samples that are essentially free from external perturbations such as impurities, solvents, and counterions. When combined with laser light sources, this approach facilitates the high‐precision determination of electronic, vibrational, and rotational energy levels, consequently revealing intrinsic molecular structures, bonding characteristics, and photodynamics. A major advancement in this area has been the development of supersonic jet expansion, which enables the preparation of cryogenically cooled gas‐phase species [1, 2]. For example, the supersonic jet of NO_2_, reported by Smalley et al., was estimated to have a rotational temperature as low as ∼3 K [1]. This cryogenic cooling effectively narrowed the initial state distribution and has significantly facilitated unambiguous spectroscopic assignments. Although supersonic jet spectroscopy remains a powerful method in modern gas‐phase spectroscopy, it has intrinsic limitations; because it requires gases or vapors to be seeded into a carrier gas prior to expansion into vacuum, its applicability is largely restricted to volatile compounds. Although several methods, such as thermal vaporization and laser desorption, have broadened the range of accessible analytes, the chemical scope of these techniques remains far from comprehensive.

**FIGURE 1:**
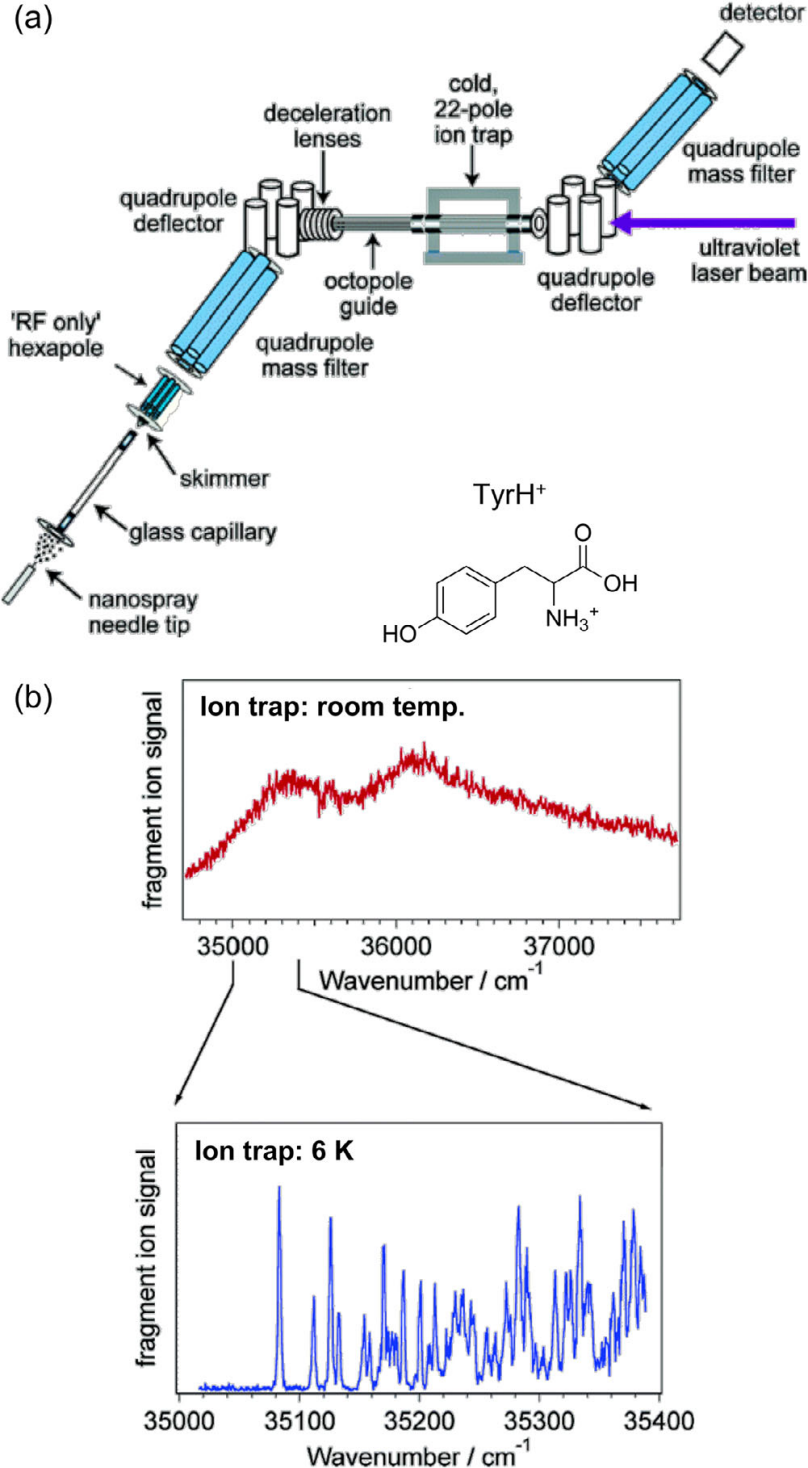
(a) Schematic view of the combined ESI‐CIT spectrometer. (b) Electronic excitation (photodissociation) spectra of protonated tyrosine (TyrH^+^; inset) in uncooled (room temperature) and cooled (ion trap at 6 K) conditions. Adapted with permission from Ref. [3].

A seminal breakthrough was reported in 2006 by Boyarkin and Rizzo, who combined electrospray ionization (ESI) with cryogenic ion trapping (CIT) for electronic spectroscopy studies [3]. This integration, as shown in Figure 1a, enabled the recording of the electronic spectrum of protonated tyrosine with markedly enhanced resolution. The spectrum exhibited clearly separated vibronic peaks [3], in sharp contrast to the room‐temperature measurement (Figure 1b), owing to the narrow distribution of the initial states. ESI is now widely recognized as the gold standard for mass spectrometry because it allows a wide range of molecules to be studied, regardless of volatility. Moreover, in principle, CIT can be coupled with other ionization methods. For these reasons, CIT‐based spectroscopic techniques have dramatically expanded the scope of cold gas‐phase spectroscopy and are strong candidates for a central role in the next generation of this field.

### 1.2 Focus of This Review: Electronic Excitation Spectroscopy With Cryogenic Ion Traps

Following these advances, CIT spectroscopy has spread rapidly over the past decade and now encompasses a broad range of gas‐phase investigations. Regarding the vibrational aspects, messenger‐tag‐assisted infrared (IR) action spectroscopy has been one of the most intensively used approaches in recent years [4, 5, 6, 7], offering high capabilities for structural determination and distinctive vibrational fingerprints that clearly identify binding motifs, proton locations, and conformer populations across diverse molecular systems [4, 5, 6, 7]. On the other hand, electronic (ultraviolet–visible (UV–Vis)) spectroscopy serves as another major aspect of gas‐phase spectroscopic studies. It provides the precise energies of vibronic states and, in favorable cases, accurate bond dissociation energies (via threshold photodissociation), while also offering insight into the photoexcited‐state dynamics of target ions. These capabilities, coupled with advances in the above‐described CIT techniques, make gas‐phase UV–Vis spectroscopy a sensitive probe of bonding characteristics and/or photochemical functions in various ions, including protonated carbocations, host–guest complexes, biologically relevant ions, metal‐containing clusters, and short‐lived chemical intermediates.

Accordingly, this review focuses on electronic excitation spectroscopy using cryogenic ion traps, with an emphasis on recent contributions. Although photoelectron spectroscopy has also yielded numerous studies revealing the electronic structures and dynamics of anionic species in the CIT [8, 9], the relevant literature has been reviewed elsewhere and is outside our main focus. Section 2 summarizes experimental schemes, ranging from established approaches to newly emerging methodologies. Section 3 presents benchmark case studies, rather than a comprehensive survey, to highlight the unique features of this technique. Our goal is to provide a concise overview of representative examples showing how CIT‐based electronic excitation spectroscopy can elucidate the intrinsic structure, bonding, and excited‐state dynamics across nonvolatile and structurally complex molecular systems spanning various fields, including biochemistry, catalysis, and materials science.

## 2 Key Techniques for Cryogenic Ion‐Trap Spectroscopy

### 2.1 Typical Setups for Cryogenic Ion Trapping

**FIGURE 2:**
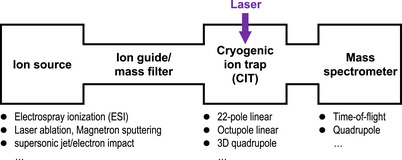
Schematic setup of a CIT‐based spectrometer.

Figure 2 schematically depicts the setup for CIT spectroscopy. Target ions are most often prepared via ESI [8], a versatile method that transfers various species in a solution into the gaseous phase with minimal fragmentation. Other ion sources reported in the literature include laser ablation or magnetron‐sputtering sources for generating naked metal‐cluster ions [10, 11, 12, 13] and combined supersonic‐jet/electron‐impact sources for generating molecular cluster ions [14, 15]. The generated ions are often mass‐separated (most typically by quadrupole mass filter) and injected into a CIT operated at ∼4 K using a closed‐cycle helium cryostat. In the trap, collisional thermalization with cold helium buffer gas quenches rotational and vibrational excitations. Regarding the trap geometries, original work by Boyarkin and Rizzo, as well as several other researchers, used a 22‐pole linear ion trap [3, 16, 17, 18], following the pioneering work on cold ion–molecule reactions by Gerlich et al., [19]. Additionally, reduced‐multipole (e.g., octupole) linear traps [20, 21, 22, 23, 24, 25, 26, 27], ring‐electrode traps [6], and three‐dimensional quadrupole ion traps (3D‐QITs) [28, 29, 30, 31, 32, 33, 34, 35, 36, 37, 38], which are often simpler to operate, have been utilized for cryogenic ion spectroscopy. Notably, a 3D‐QIT has been adopted in earlier photoelectron experiments [39]. Linear traps are typically more suitable for ion loading, whereas 3D‐QITs confine the ion bunch to a smaller volume, improving spatial overlap with the laser. Although some early reports noted severe RF heating in 3D‐QITs [34], subsequent studies have shown that, under appropriate conditions, they provide sufficient cooling for high‐quality electronic spectroscopy [35, 36]. For quadrupole (linear and 3D) traps, applying an auxiliary radio frequency (RF) voltage enables *m*/*z*‐selective ion ejection [40, 41, 42], facilitating hole‐burning (HB) experiments (particularly in the UV–UV scheme), as discussed in Section 2.3.1. The stored and cooled ions are irradiated with a laser output, and the obtained photofragment ions and remaining parent ions are detected using a mass spectrometer (most typically time‐of‐flight or quadrupole type) for any action spectroscopy, as discussed in Section 2.2.

Numerous practical improvements to trap configurations, electrode geometries, and RF voltage waveforms have been proposed and demonstrated. For example, a ring‐electrode‐assisted 22‐pole trap reported by Jusko et al., [43]. enabled the fine kinetic energy control of slow, cold ions. Polfer et al. developed a mass‐selective ‘two‐dimensional linear trap’ (or originally, ‘rectilinear ion trap’ [44]) for cryogenic spectroscopy [45] that did not require a separate mass analyzer and was consequently more compact. Asmis et al. developed split‐ring electrode ion traps for the action spectroscopy of single nanoparticles weighing several hundred MDa [46]. Garand et al. implemented digital square‐wave RF voltages for linear traps [47], allowing both microsolvation and mass filtering to be performed in a single trap prior to spectroscopic probing. These techniques are expected to make ion trap spectroscopy more versatile and accessible.

### 2.2 Typical Schemes of Action Spectroscopy

#### 2.2.1 Photofragmentation

In ion traps, the number density of stored ions is intrinsically limited by the space‐charge effect. Consequently, it is impractical to measure the direct absorbance spectra, that is, the ratio of fluence between incident and transmitted light, which is the most common approach in spectroscopy. Instead, ion spectroscopy is typically employed as an action spectroscopy approach, in which the “action” induced by photon absorption is monitored, as schematically summarized in Table 1. Among these methods, photodissociation (PD) (or photofragmentation) spectroscopy is the most widely used. In this approach, photon absorption triggers cleavage of the bond with the target ion ((A−B)^+^).

(1) $${\left(A-B\right)}^{+}\overset{\mathrm{hv}}{\to }\;A^{+}+B$$

**TABLE 1:** Typical action spectroscopic techniques for ions in cryogenic ion traps.

Spectroscopy	Probed action
Photodissociation spectroscopy	Photofragment
	(A^+^ in Equation 1)
	Evaporation of inert tag molecules
	(M^+^ in Equation 2)
Photodetachment spectroscopy	Total photoelectron yield
	(e^−^ in Equation 3)
Laser‐induced fluorescence spectroscopy	Fluorescence
	(*hν*’ in Equation 4)
Leak‐out spectroscopy	Translationally hot target ions
	escaped from CIT
	(M^+^ in Equation 5)
Cavity ring‐down spectroscopy	Ring‐down rate of intracavity light^a^

^a^ *Note*: because it provides a direct absorption measurement of target ions, this is not an ‘action’ in the strict sense.

The resulting change in ion abundance, that is, either the appearance of fragment (A^+^) or depletion of parent ((A−B)^+^), is recorded via mass spectrometry as an alternative to absorbance. When the dissociation efficiency following photoabsorption is unity, the resulting spectra are equivalent to the true absorption spectrum.

#### 2.2.2 Evaporation of Inert Tag Molecules

The laser light used for electronic spectroscopy (UV–Vis) typically affords sufficient photon energy to cleave the bonds in isolated ions. However, when ions have many vibrational degrees of freedom, the photodissociation quantum yield may fall below unity because the internal energy gained upon excitation is rapidly redistributed and competes with collisional cooling by the buffer gas molecules, as recently demonstrated for silver cluster cations [48]. To address this, inert “tag” (or “messenger”) molecules (He, Ne, Ar, H_2_, N_2_, etc.; M_tag_) are weakly attached to the target ions (M^+^). The very small binding energies of the tagged molecules provide a facile, well‐defined dissociation channel.

(2) $$M^{+}\cdots M_{\text{tag}}\;\overset{\mathrm{hv}}{\to }\;M^{+}+M_{\text{tag}}$$

After photoabsorption, the tag molecule evaporates, producing a clear action signal while minimally perturbing the electronic states of the chromophore. Spectral shifts caused by tagging are typically limited to a few cm^−1^. However, it should be noted that some studies have pointed out the non‐negligible effects of tag molecules on the observed vibronic profiles [49, 50].

#### 2.2.3 Other Methods

Several additional action‐detection schemes have been developed for ion spectroscopy. For anions, photodetachment spectroscopy has been used to monitor the total photoelectron yield as a function of excitation wavelength [8].

(3) $$M^{-}\;\overset{hv}{\to }\;M^{0}+e^{-}$$

This provides a powerful probe for bound excited states embedded in the energy continuum of the photodetached states. When combined with the resonant two‐photon detachment (R2PD) scheme [8], it further affords excited‐state energy levels below the electron‐detachment threshold, as exemplified by the characterization of dipole‐bound states.

Another scheme includes the detection of laser‐induced fluorescence (LIF) [51, 52, 53, 54, 55],

(4) $$M^{+}\;\overset{\mathrm{hv}}{\to }\;{\left(M^{+}\right)}^{*}\;\to M^{+}+h\nu^{\prime }$$

which has a decades‐long history and is well known to offer complementary information to other action schemes, such as the vibrational states of the electronic ground state (via dispersed fluorescence detection) and fluorescence lifetimes, although most reports are limited to small ions and highly fluorescent samples [56, 57, 58, 59].

A more recent approach is leak‐out spectroscopy (LOS), which was first demonstrated for vibrational excitation [60] and has now been extended to electronic spectroscopy [61]. In LOS, photoexcited target ions undergo V‐T energy transfer via collision with buffer gas molecules, acquiring sufficient kinetic energy to leave the trap potential; the escaped ions are then detected via mass spectrometry.

(5) $$M^{+}\;\overset{\mathrm{hv}}{\to }\;{\left(M^{+}\right)}^{*}\;\overset{V-T}{\to }M^{+}\left(\text{translationally hot}\right)$$

Because it does not rely on fragmentation or tagging, LOS is, in principle, broadly applicable to a wide range of ions and even low‐lying electronic excited states in the near‐infrared (NIR) and visible regions. Finally, direct absorption by trapped ions can be measured using cavity ring‐down spectroscopy (CRDS) [62]. In this approach, a high‐finesse optical cavity is constructed around a (linear) ion trap, and the ring‐down rate of the intracavity light, shortened by absorption by ions, is monitored. The resulting decay constant is linearly proportional to the photoabsorption cross‐section, enabling ultrahigh‐sensitivity measurements.

### 2.3 Advanced Spectroscopic Techniques

#### 2.3.1 Isomer‐Selective Spectroscopy

This section introduces advanced CIT‐based spectroscopic techniques that extract information beyond band positions/oscillator strengths. A key advantage of cryogenic gas‐phase spectroscopy is that the narrow spectral lines enable the application of a HB scheme to distinguish a single isomer (conformer) within a congested electronic spectrum. In a two‐color UV‐UV HB experiment, two lasers, a burn (pump) pulse and a probe pulse, are used. The probe wavenumber is fixed at the selected vibronic transition of the target ion, which generates a fragmented ion signal. A burn pulse is introduced before the probe pulse and scanned. When the burn pulse excites the target ion, it creates a “population hole” in the ground state; consequently, the probe‐induced fragment signal is depleted. Recording this depletion as a function of the burn wavenumber yields a dip spectrum that reflects only the single isomer associated with the selected probe transition, thereby achieving isomer‐selective spectroscopy. Practically, the fragment ions generated by the burn pulse must be removed before the probe shot, and several implementations have been demonstrated for this purpose [41, 63, 64]. Electronic (UV‐UV) HB for CIT‐stored ions was first demonstrated by Choi et al. for alkali metal‐crown ether complexes [63]. Subsequently, it has been applied broadly, especially to flexible ions such as host–guest complexes and peptides/protein ions [65], where conformer‐resolved spectra are essential for unambiguous assignments. It is worth noting that isomer‐selective spectroscopy can also be achieved by coupling ion traps with ion‐mobility mass spectrometry (IM‐MS) [38, 66, 67]; detailed instrumentation and applications have been reviewed elsewhere [68, 69].

#### 2.3.2 Ultrafast Spectroscopy

Ultrafast pump‐probe spectroscopy is one of the most extensively explored methodologies in modern spectroscopy, providing direct access to the time evolution of wave packets on photoexcited potential‐energy surfaces. In the context of ion spectroscopy, applications to electronic excitation (e.g., photodissociation) spectroscopy remain relatively limited compared to the numerous anion photoelectron studies [70, 71]. In the pump‐probe electronic excitation of ions in CITs, both pulses can contribute to excitation and subsequent photodissociation. Depending on the probe wavenumber and topology of the excited‐state surfaces, the probe pulse may cause enhanced or new photofragments via two‐color sequential absorption or decreased fragment yield by depleting the excited‐state population (e.g., via ground‐state recovery). In all cases, monitoring the fragment signals as a function of pump‐probe delay yields state‐specific lifetimes and wave‐packet dynamics, including the surpassing of small excited‐state barriers and following nonadiabatic decays (internal conversions (ICs)/intersystem crossings (ISCs)) [72]. To date, demonstrations have focused largely on bio‐related ions, including protonated amino acids and their micro‐hydrated complexes, protonated retinal [73], and fluorescent protein chromophores [27].

#### 2.3.3 Other Methods

Several additional advanced schemes for ion‐trap spectroscopy have been developed. Recently, Kim et al. integrated UVPD spectroscopy of ions in CITs with circular dichroism (CD) spectroscopy, called UVPD‐CD spectroscopy, to record the state‐ and conformer‐selective circular dichroism of gaseous ions [74, 75]. In this approach, the CD spectrum is obtained as the difference in the photofragment yield induced by left‐ and right‐handed circularly polarized (LCP/RCP) UV pulses. A multiple laser shot technique is used to improve the signal‐to‐noise (S/N) ratio. In this method, ions are irradiated by a sequence of several laser pulses within the CIT prior to extraction and mass analysis. The application to protonated phenylalanine and phenylalanylalanine demonstrated that combining UVPD‐CD and HB spectroscopic techniques (see Section 2.3.1) yields state‐ and conformer‐specific CD responses.

Another advanced method described herein involves the measurement of not only fragment yields but also angular distributions and kinetic‐energy release (KER) via photofragment velocity‐map imaging (VMI) [32, 76]. In these instruments, collisionally cooled ions are extracted from the CIT, mass‐selected (typically by time‐of‐flight), and intersected with a polarized laser pulse within a carefully designed electrostatic lens assembly for VMI. The resulting photofragments are accelerated onto a position‐sensitive microchannel plate coupled with a phosphor screen, and transient images are recorded using a charge‐coupled device (CCD) camera. Electrode geometries are finely optimized, often based on ion trajectory simulations, to achieve a better resolution for CIT‐cooled ion beams. VMI has been demonstrated using several small molecular ions (such as Br_2_^+^ and N_2_O^+^) [32, 76, 77, 78, 79], metal‐containing ions (such as Al_2_^+^ and MgI^+^) [80, 81], and ionic complexes (such as [O_2_–H_2_O]^+^) [82]. More recently, the application to coincidence measurements of photofragment ions with neutral counterparts [83] and photoelectrons [84] has been reported.

## 3 Case Studies

### 3.1 Carbocations and Protonated Hydrocarbons

**FIGURE 3:**
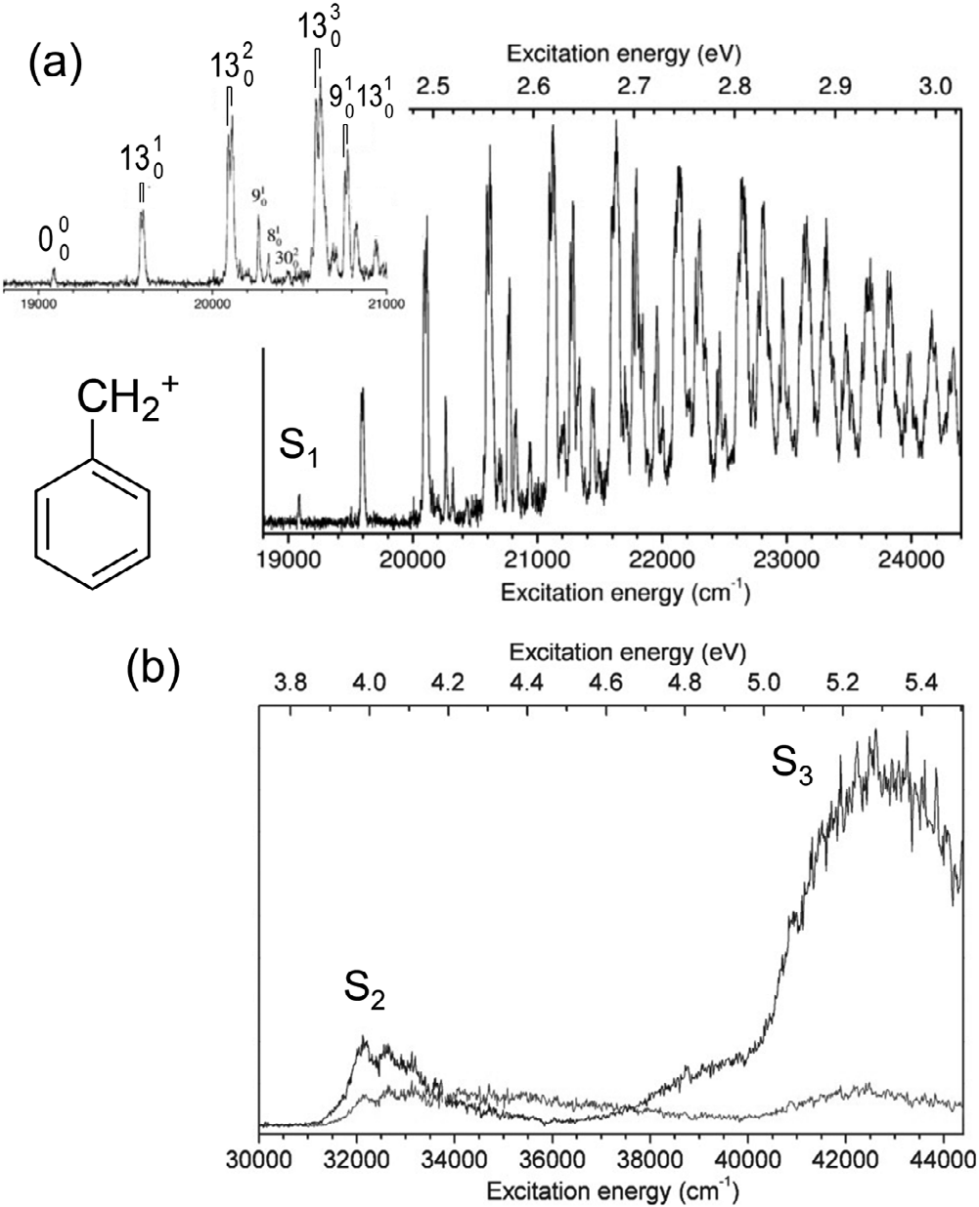
Electronic photodissociation spectra of a benzylium cation ([C_6_H_5_─CH_2_]^+^; inset) recorded in the (a) visible region (18800–24400 cm^−1^) and (b) ultraviolet region (30000–44500 cm^−1^). Spectrum (a) was recorded by detecting C_5_H_5_^+^ fragments. The inset depicts an expanded view of the 19000–21000 cm^−1^ region, with eye‐guides indicating several splitting peaks. For (b), the black and blue spectra were recorded by detecting different fragments (black: C_5_H_5_^+^, blue: C_3_H_3_^+^). Adapted with permission from Ref. [85].

This section focuses on the application of CIT spectroscopy to several benchmark molecular systems as case studies. As prototypical systems, small carbocations and protonated aromatics serve as good models for demonstrating the ability of electronic excitation spectroscopy under cryogenic conditions. A representative example is the UV–Vis PD spectroscopy of phenylalkyl carbocations [C_6_H_5_─R]^+^ (R ═ CH_2_ and C_2_H_4_) [85], reported by Jouvet et al.; spectra for R = CH_2_ are shown in Figure 3. The S_1_←S_0_ bands in the visible region (18800–24400 cm^−1^; Figure 3a) exhibit a well‐resolved vibronic structure that is significantly narrower than in argon‐tagged measurements, indicative of effective collisional cooling in the CIT. Vibrational assignments for this progression can be found in Refs. [85] and [86]. Notably, the improved resolution due to the utilization of CITs revealed a fine splitting of the *ν*_13_ (ring deformation) progressions with a spacing of ∼504 cm^−1^, attributed to Fermi resonance with combined out‐of‐plane modes. In contrast, the UV bands (30000–44500 cm^−1^; Figure 3b), corresponding to S_2,3_←S_0_ transitions, exhibited a considerably broadened profile even under the cold gas‐phase conditions. This reflects substantial structural changes and/or fast nonradiative decay dynamics on higher‐excited‐state surfaces. Similar broadening has been reported for the S_1_←S_0_ transition of protonated benzene (C_6_H_6_·H^+^) and toluene (C_6_H_5_CH_3_·H^+^) [87] under cryogenic conditions, emphasizing that the electronic band profiles of CIT‐cooled ions can provide dynamical information in addition to structural fingerprints.

More broadly, the electronic spectroscopy of carbocations is often studied in terms of astrochemical interest. Recent studies have provided clear Vis‐NIR band assignments for polyaromatic hydrocarbons (PAHs) [88, 89, 90], carbon chains/rings [91, 92, 93, 94], and C_60_^+^ [95] in CITs. These gas‐phase benchmarks provide a laboratory reference for diffuse interstellar bands (DIBs) [96], in several cases identifying plausible candidates and, in the case of C_60_^+^, affording a definitive assignment [95].

### 3.2 Ionic Organic Dyes

**FIGURE 4:**
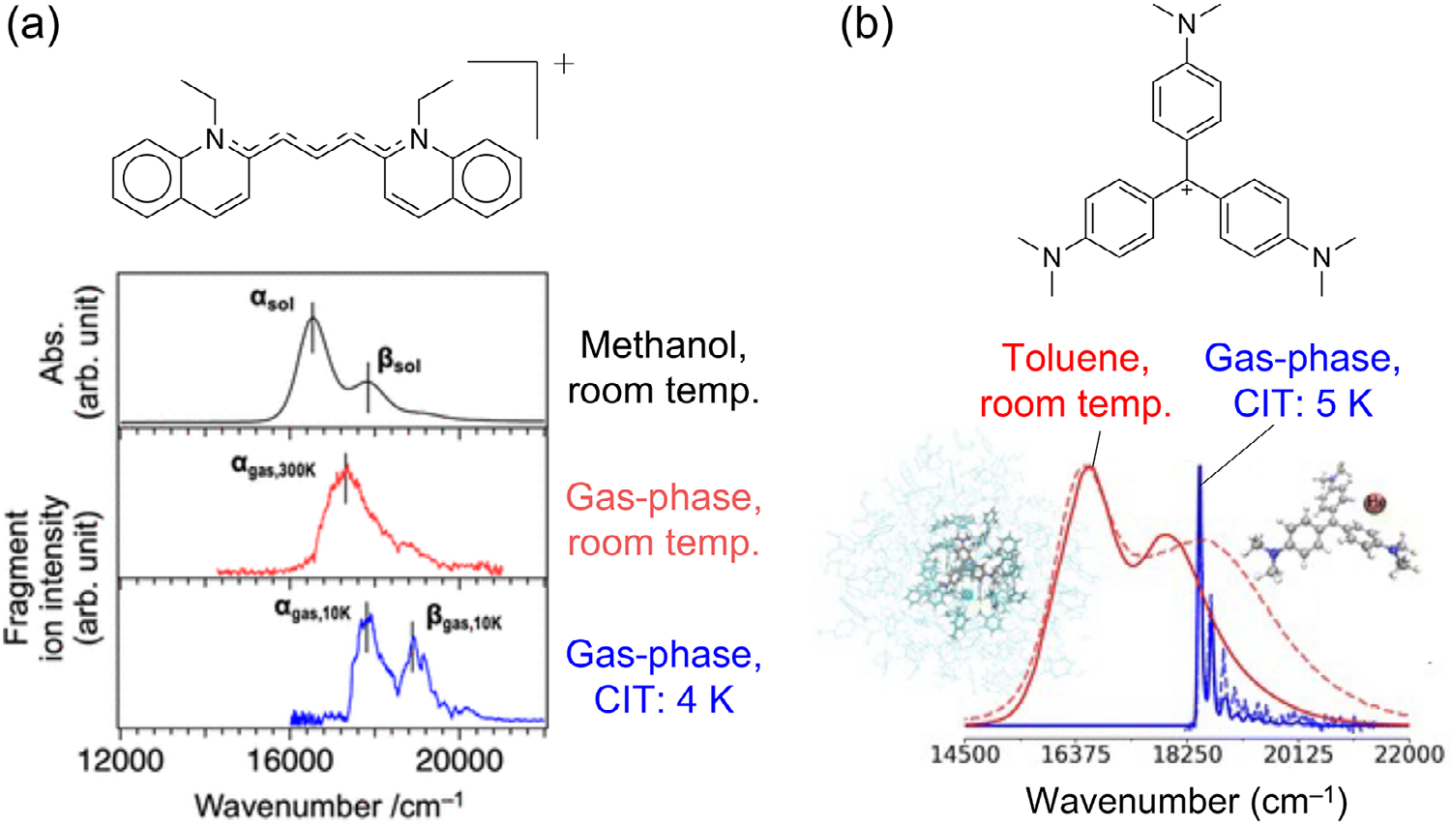
(a) Comparison of the electronic spectra of carbocyanine dye (pinacyanol) recorded in the room‐temperature methanol solution, room‐temperature gas‐phase, and cold (CIT: 4 K) gas‐phase. (b) Comparison of the electronic spectra of crystal violet cations recorded in the room‐temperature toluene solution and cold (CIT: 5 K) gas‐phase. The dashed lines denote experimental spectra, whereas the solid lines represent simulated ones. Adapted with permission from Ref. [97] for (a) and Ref. [98] for (b).

**FIGURE 5:**
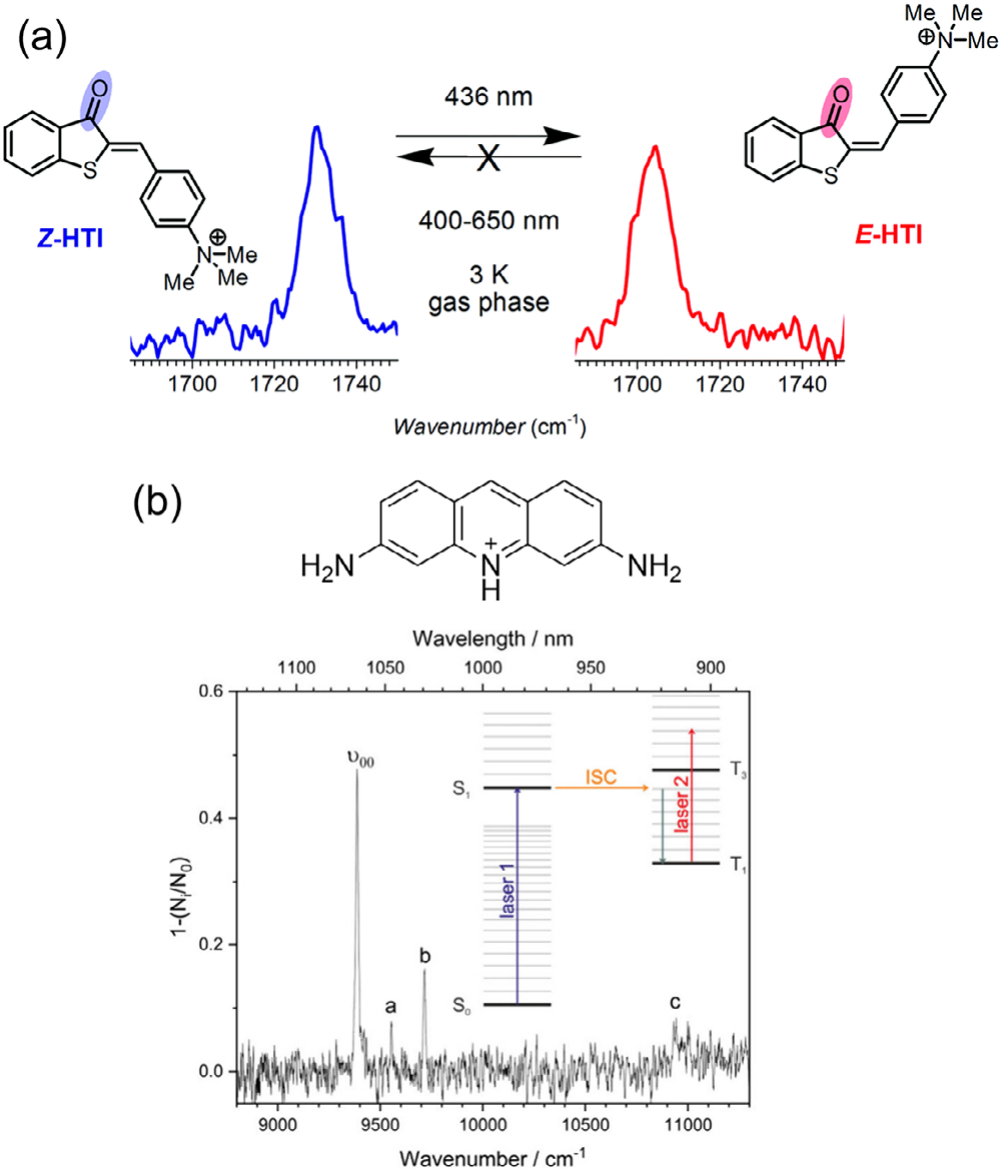
(a) Isomer‐specific vibrational spectra (C═O stretching region) of *Z* and *E* isomers of hemithioindigo derivatives. The E isomer was generated via photoisomerization of the corresponding *Z* isomer inside the CIT. (b) State‐specific electronic spectra of T_1_‐state protonated proflavine, formed via ISC from the photoexcited S_1_ state inside the CIT. Adapted with permission from Ref. [106] for (a) and Ref. [107] for (b).

Organic conjugated dyes have long been intensively studied as spectroscopic targets because their large absorption and fluorescence cross sections (intense ππ* bands) make them ideal test systems for new spectroscopic methodologies. Recent work shows that their ππ* transition energies and vibronic profiles respond sensitively to environmental conditions (e.g., cold gas‐phase (CIT) vs. room‐temperature solution) as well as conformational changes, as exemplified for carbocyanine dyes (Figure 4a) [97], crystal violet cations (Figure 4b) [98], and others [99, 100, 101, 102, 103, 104, 105]. Beyond static band shifts and narrowing, many dyes exhibit distinct nonadiabatic decay pathways, notably *trans*–*cis* photoisomerization along conjugated double bonds and ISC, enabled by small S_1_–T_1_ energy gaps. In this regard, the direct characterization of the resulting photoproducts in a solvent‐free environment is of interest. Roithová et al. achieved the isomer‐specific identification (both from electronic and vibrational spectroscopies) of the *E* isomer of hemithioindigo derivatives generated via photoisomerization of the corresponding *Z* isomer inside the CIT (Figure 5a) [106]. Strikingly, only *Z* → *E* conversion was observed in the gas‐phase, in sharp contrast to the solution, where both directions are accessible; this highlights the strong effect of solvation for excited‐potential profiles. More recently, Kappes et al. reported the electronic spectra of protonated proflavine and acridine orange in T_1_ states, formed via ISC from the photoexcited S_1_ states inside the trap (Figure 5b) [107]. These measurements, combined with fluorescence and phosphorescence spectroscopies in the solid neon matrix, enabled vibronic‐level assignments for the S_1_↔S_0_, T_3_←T_1_, and T_1_→S_0_ transitions, which are benchmarks that remain challenging for standard computational methods to reproduce quantitatively.

### 3.3 Host–Guest Complex Ions

**FIGURE 6:**
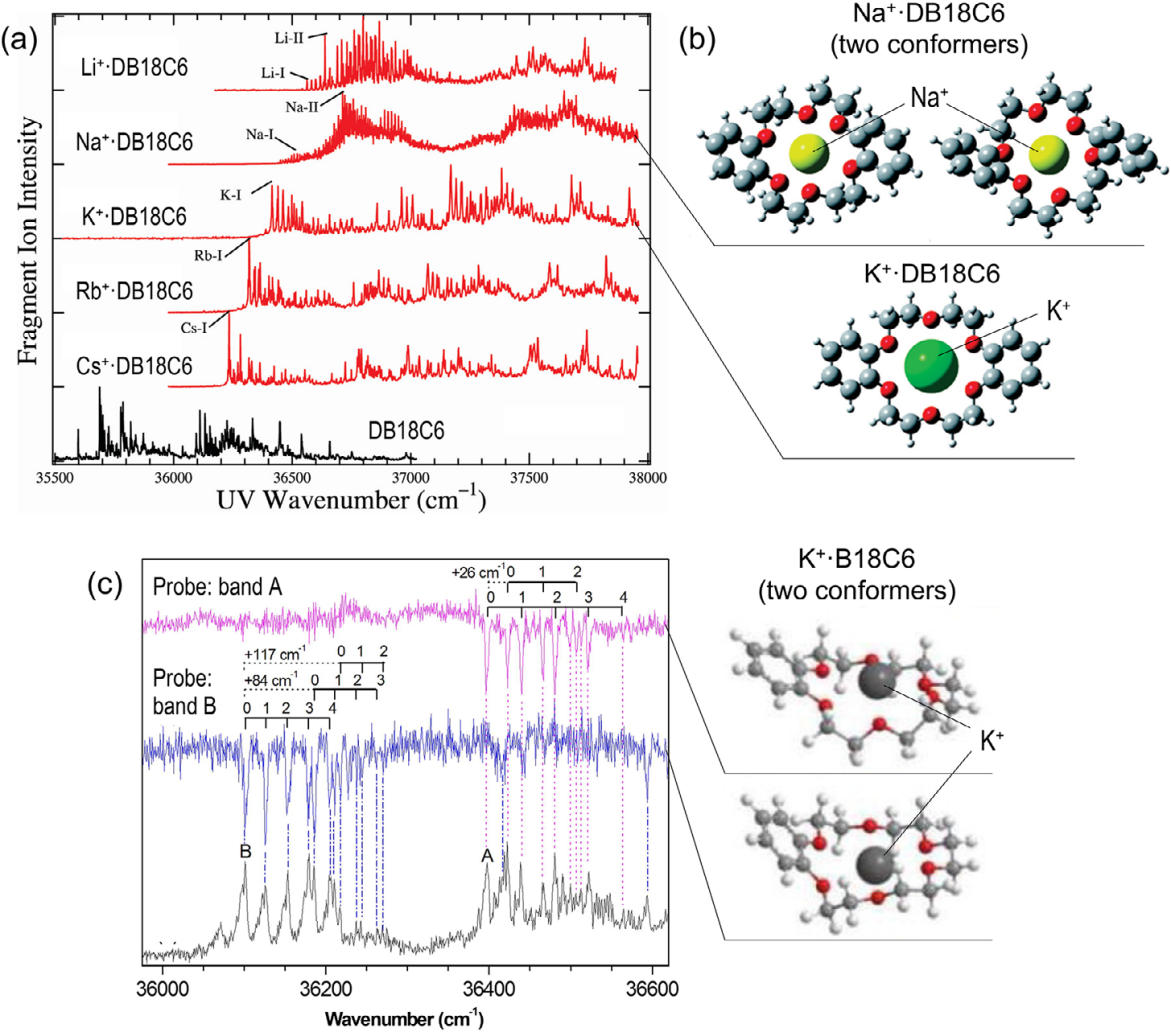
(a) UVPD spectra of M^+^·DB18C6 (M = Ki, Na, K, Rb, and Cs) in comparison with the LIF excitation spectrum of monomeric DB18C6 (recorded in a supersonic jet). (b) Structural difference between Na^+^·DB18C6 and K^+^·DB18C6 (calculated at M05‐2X/6‐31+G(d)). (c) UV–UV HB spectra of K^+^·B18C6 (pink and blue) in comparison with the UVPD spectrum (gray). The peak notations, A and B, represent the probe laser wavenumbers for the HB measurements. The corresponding structures of K^+^·B18C6 (calculated at M05‐2X/6‐31+G(d)) are also shown. Adapted with permission from Ref. [108] for (a) and (b), and Ref. [109] for (c).

Vibronic patterns often provide a sensitive probe for slight conformational differences in flexible molecules, and some striking demonstrations have been found in host–guest complexes. A seminal study by Inokuchi et al. in 2011 [108] reported the vibronically resolved UVPD spectra of dibenzo‐18‐crown‐6‐ether–alkali‐metal complexes, M^+^·DB18C6 with M = Li, Na, K, Rb, Cs (Figure 6a). The vibronic patterns changed drastically between M = Na and K, providing a spectroscopic marker of different encapsulation motifs (Figure 6b); for M = K, the crown part maintained its symmetrical conformation, whereas that in M = Na was slightly twisted. Soon after, Kim et al. first reported the UV–UV HB spectra of the K^+^–benzo‐18‐crown‐6‐ether complex (K^+^·B18C6) [109], revealing the presence of multiple conformational isomers of this complex with distinctly different vibronic structures, arising from the flexible framework of the host molecule (Figure 6c).

**FIGURE 7:**
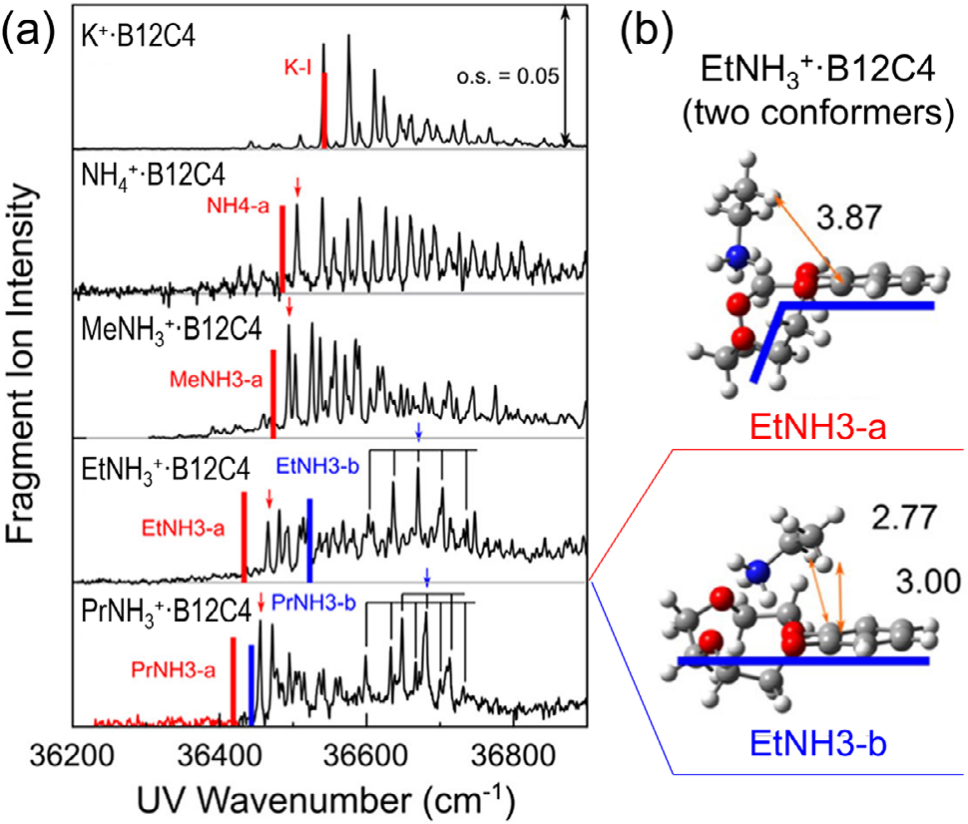
(a) UVPD spectra of RNH_3_^+^·B12C4 (R = H, CH_3_, C_2_H_5_ (Et), C_3_H_7_) in comparison with that of K^+^·B12C4. The red and blue bars indicate the calculated electronic‐excitation energy (at the (TD‐)ωB97X‐D/6‐311++G(d,p) level). (b) Structures of two conformers found for EtNH_3_^+^·B12C4; bond lengths are presented in Å and blue bars are eye‐guides indicating the positional relationship between the crown cavity and the benzene ring. Adapted with permission from Ref. [111].

More recent examples include the extension of this approach to crown‐ether–ammonium ion complexes [110, 111, 112]. Figure 7a presents the UV spectra for benzo‐12‐crown‐4 (B12C4)–ammonium complexes, RNH_3_^+^·B12C4 with R = H, CH_3_, C_2_H_5_, C_3_H_7_ [111]. The vibronic envelopes resemble those of the alkali‐metal analog K^+^·B12C4, with a slight blueshift of the origin bands; empirically, this is explained by a similar host framework with weaker interaction as the alkyl chain lengthens. Strikingly, for R = C_2_H_5_ and C_3_H_7,_ an additional vibronic progression appears in higher wavenumber regions (>36600 cm^−1^), indicating the emergence of new conformers. With the aid of IR–UV double resonance spectroscopy and DFT calculations, these new isomers were assigned to structures partially stabilized by C─H···π type interactions owing to the longer alkyl chains, in addition to the primary N─H···O hydrogen bonds between the –NH_3_^+^ group and the crown cavity (Figure 7b). A series of these studies demonstrated that CIT‐based electronic spectroscopy is a highly sensitive probe for small conformational changes discerned in flexible host–guest molecules. Recent extensions include other hosts such as calixarenes [35, 113, 114], cryptands [115], and spherands [116], as well as combined studies with IM‐MS for larger dibenzo‐crown‐alkali‐metal complexes [117], enabling analyses of isomerization dynamics across increasingly complex systems.

### 3.4 Microhydrated Ions

**FIGURE 8:**
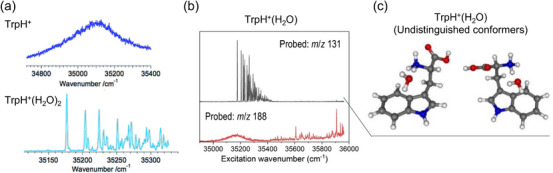
(a) UVPD spectra of TrpH^+^ and TrpH^+^(H_2_O)_2_. (b) UVPD spectra of TrpH^+^(H_2_O) recorded by probing different photofragment channels: *m*/*z* 131 (black) and 188 (red). (c) Calculated structures (B3LYP‐D3BJ/cc‐pVTZ) of TrpH^+^(H_2_O) that yield the vibronic spectrum in (b) with an *m*/*z* 131 fragment. Adapted with permission from Ref. [123] for (a) and Ref. [127] for (b) and (c).

Microhydration is one of the central themes in gas‐phase spectroscopy because coupling it with mass spectrometric techniques enables the stepwise control of the number of solvent (H_2_O) molecules with single‐molecule‐level precision. These systems have been intensively studied using IR spectroscopy because the O–H stretching spectral patterns clearly reflect the evolution of hydrogen‐bond networks with the number of solvent molecules [118, 119, 120, 121, 122]. In the context of electronic spectroscopy, particularly in combination with CIT, an early benchmark was the study of protonated tryptophan (TrpH^+^) by Rizzo et al. in 2006 [123]. Bare TrpH^+^ exhibits a featureless, broadened electronic spectrum [3], consistent with the ultrafast decay observed in femtosecond time‐resolved measurements [124] and rationalized by intensive computational studies on excited‐state ππ*/πσ* surfaces [125, 126]. In sharp contrast, the dihydrated cluster TrpH^+^(H_2_O)_2_ possesses well‐resolved vibronic bands, as shown in Figure 8a [123], demonstrating the drastic effects of hydration on the excited‐state relaxation. More recently, Grégoire et al. revealed the conformer‐dependent photodissociation dynamics in monohydrated TrpH^+^(H_2_O) [127]; different conformers yielded different fragment patterns and excited‐state lifetimes, as shown in Figure 8b. In a mechanistic perspective, they revealed that the H_2_O molecule bridging the ammonium and indole rings hinders the barrierless excited‐state proton transfer (ESPT) by forming hydrogen bond networks, which lengthens the S_1_ lifetime and yields a sharp vibronic profile in the UVPD spectrum (Figure 8c). The hydration‐induced suppression of ESPT is not a system‐specific phenomenon and has also been observed for other molecules, including protonated dopamine [128, 129], again converting lifetime‐limited broad UV bands into resolved vibronic structures with three H_2_O molecules as microsolvents. Similar microsolvation‐induced modulations of the electronic states of CIT‐cooled gas‐phase ions have recently been investigated across various systems [130, 131, 132, 133, 134, 135].

### 3.5 Hypervalent Ions

**FIGURE 9:**
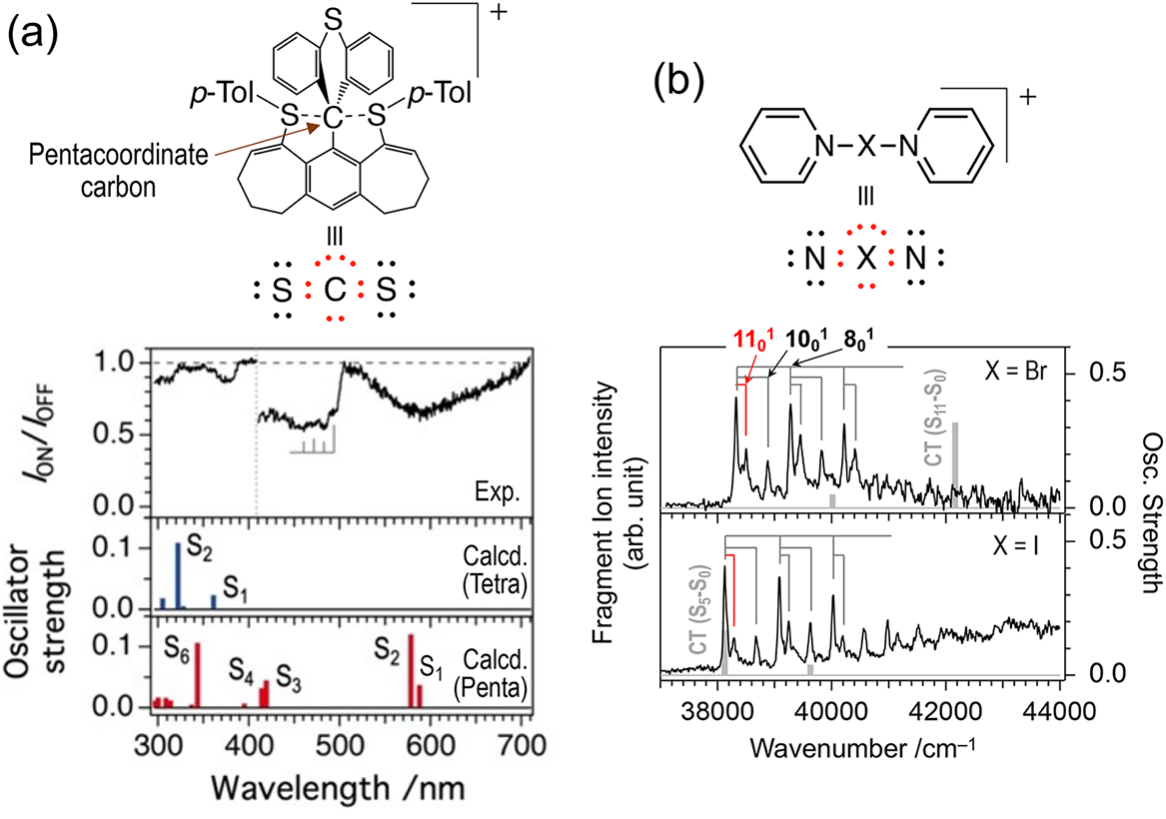
(a) Structure and UV–VisPD spectrum of the hypervalent pentacoordinate carbon compound. The bar spectra show the calculated electronic excitation energies of the target pentacoordinate compound (red) and tetracoordinate (normal valent) isomer (blue). (b) Structure and UVPD spectra of hypervalent halogen compounds, [(C_5_H_5_N)_2_X]^+^ (X = Br, I). Adapted with permission from Ref. [138] for (a) and Ref. [139] for (a).

Hypervalent (or hypercoordinate) compounds contain main group centers that formally possess more than eight valence electrons in their Lewis dot structures. Because their stability is commonly rationalized in terms of their characteristic electronic structures, most notably the three‐center four‐electron (3c–4e) bonding model, direct spectroscopic characterization of these motifs is highly insightful. While several IR spectroscopic studies on cold solid matrices have provided valuable structural benchmarks for selected hypervalent species [136, 137], gas‐phase electronic spectroscopy in CIT will offer complementary perspectives. For hypervalent carbon compounds [138], which have been of interest in relation to good models of the transition state in S_N_2 reactions, the electronic spectrum displays broad features (Figure 9a), and the absorption in the visible region is a characteristic signature of the pentacoordinate (hypervalent) form of this compound, assigned to transitions with partial 3c‐4e characteristics. Combined analysis with IM–MS and DFT calculations revealed the enhanced stability of the hypervalent form in the gas phase when compared to the single‐crystal condition. In contrast, for hypervalent halogen compounds, bispyridine halonium ([(C_5_H_5_N)_2_X]^+^ (X = Br, I)) [139], prominent vibronic progressions were discerned for bands assigned to charge‐transfer (CT) transitions that move electron density from the central halogen toward the terminal pyridine rings (Figure 9b). This was a clear indication of halogen bonds, also contributing to the linear N−X−N structure and the formal 10‐electron picture at the halogen center. The vibronically resolved band enabled quantitative extraction of the effective N−X bond force constants in the excited CT state, where the difference in this value between X = I and Br is partly associated with the degree of electron transfer upon CT excitation. These studies are expected to provide a new spectroscopic perspective on hypervalency.

### 3.6 Metal Cluster Ions

**FIGURE 10:**
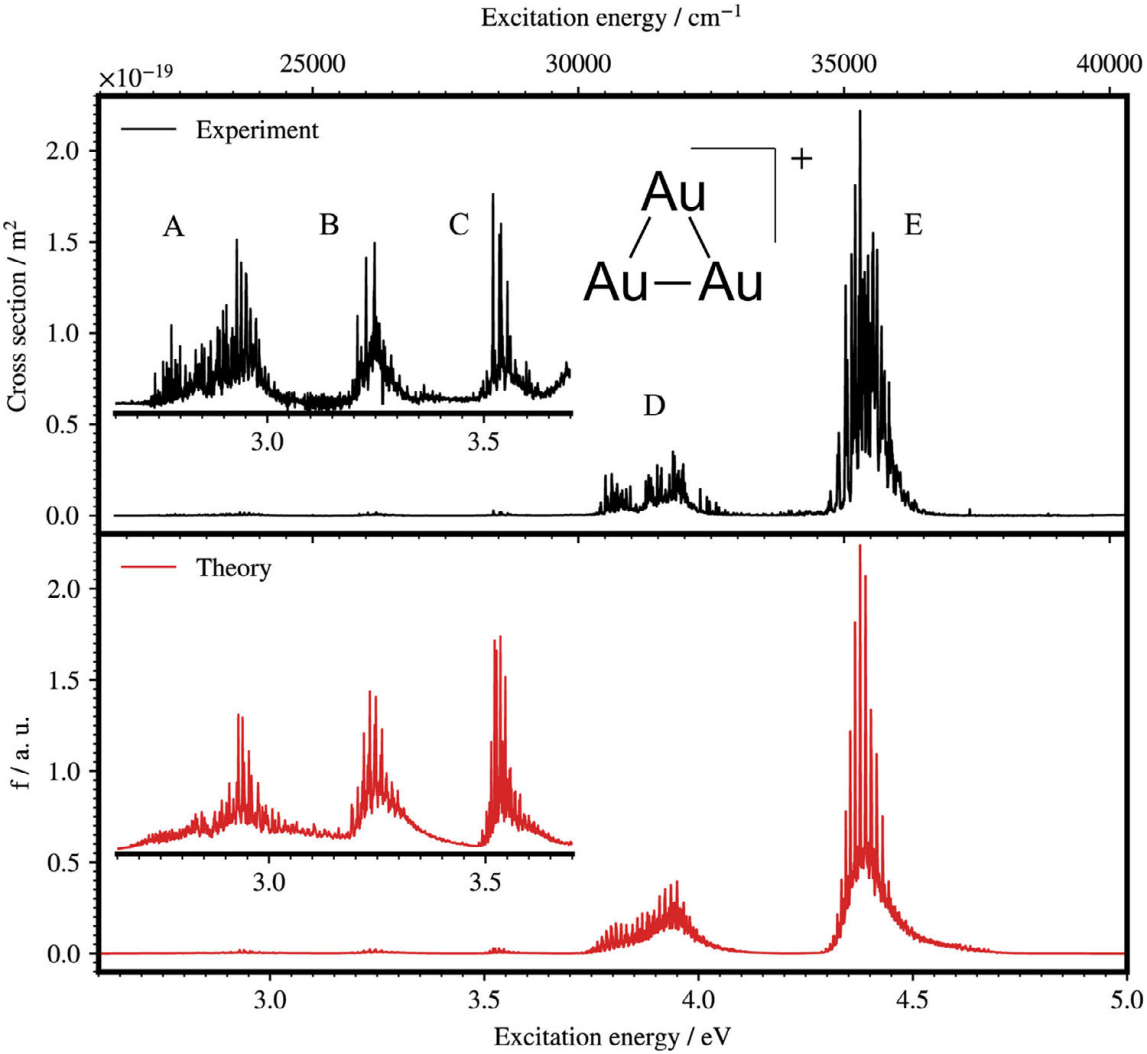
Experimental UV–VisPD spectrum of Au_3_^+^ (black) and calculated spectrum (red). Adapted with permission from Ref. [140].

Metal clusters composed of several to tens of metal atoms (in particular, transition metals) typically exhibit much more congested electronic structures than ordinary organic ions. In this context, CIT‐based ion cooling is highly effective in resolving detailed electronic states. A prominent example is the Au_3_^+^ cation with a triangular geometry, which was investigated by Mitric, Dopfer, et al., [140]. The UVPD spectra (Figure 10) resolved five distinct closely spaced vibronic bands (denoted as A–E) across the 2.7–5.0 eV range. High‐level quantum chemical calculations at the complete active space self‐consistent field (CASSCF) with a large active space reproduced these features well (Figure 10), demonstrating the essential contributions of d‐orbital excitations, a small s‐d orbital energy gap, and substantial vibronic and spin‐orbit couplings to the spectra beyond the simplified superatom descriptions. More generally, the congested excited‐state bands of transition metal clusters provide favorable conditions for threshold photodissociation measurements to determine the bond dissociation energies (BDEs) [141, 142, 143]. In this context, ion cooling by CIT sharpens the fragmentation onset and thus improves the accuracy of BDEs, as demonstrated for several metal‐containing complexes and small clusters (e.g., Fe_2_^+^).

**FIGURE 11:**
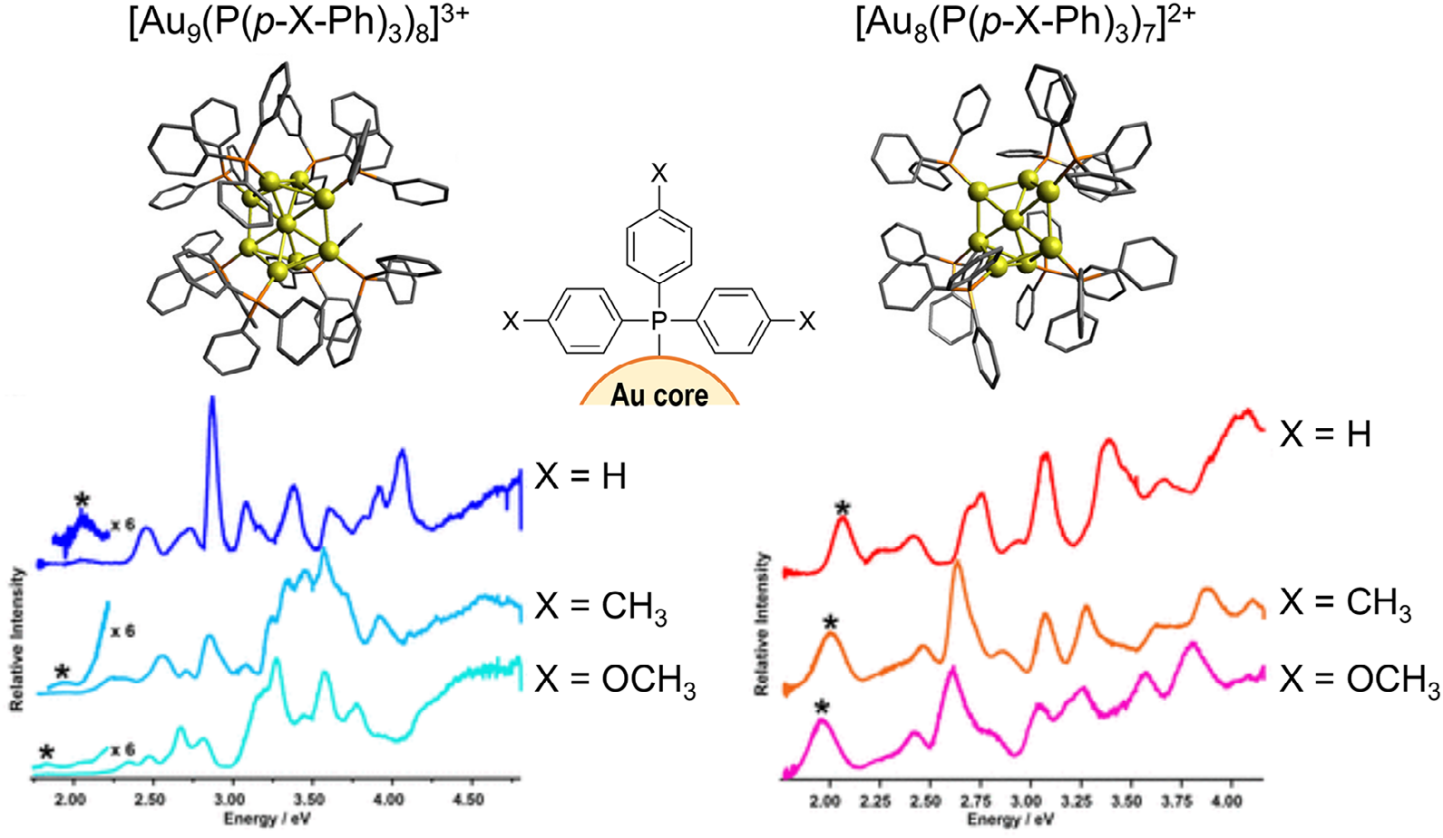
UV–VisPD spectrum of [Au_9_(P(*p*‐X‐Ph)_3_)_8_]^3+^ (left) and [Au_8_(P(*p*‐X‐Ph)_3_)_7_]^2+^ (right) (X = H, CH_3_, OCH_3_). The indicated structures are those of X = H in single crystals (H atoms are omitted for clarity). Asterisks in the spectra indicate HOMO–LUMO transition bands. Adapted with permission from Ref. [25].

Beyond the above‐described “bare” clusters, ligand‐protected metal clusters are increasingly important in catalysis, photochemistry, and materials science, where their electronic structures govern both reactivity and luminescent behavior. Johnson et al. recently reported the electronic excitation spectra of CIT‐cooled phosphine‐protected Au clusters, including [Au_9_(P(*p*‐X‐Ph)_3_)_8_]^3+^ and [Au_8_(P(*p*‐X‐Ph)_3_)_7_]^2+^ (X = H, CH_3_, OCH_3_), as shown in Figure 11a,b [25, 144]. Owing to the high spectral resolution, they revealed that cluster‐core‐localized excitations shift systematically with the electron‐donation ability of the ligands. In particular, the HOMO–LUMO transition energy exhibited a clear linear correlation with the Hammett parameters of the para‐substituted phosphine ligands, providing a direct visualization of ligand tuning on the electronic structure of the metal cluster core.

### 3.7 Ionic Chemical Intermediates Formed in Solution

The elucidation of the composition, electronic structure, and conformations of short‐lived reaction intermediates is essential for a full understanding of chemical reactions. Time‐resolved optical spectroscopy is the most prototypical and widely used method for detecting transient species in solutions. However, in practice, two factors often make such measurements challenging: the intermediates are typically formed at very low concentrations, and signals from reactants and products overlap with those of the intermediates, limiting selectivity. In this context, CIT‐based gas‐phase spectroscopy is expected to be a complementary strategy. If the species from an ongoing reaction solution can be transferred to the gas‐phase, the intermediate of interest can be spatially mass‐separated from other components and probed with high sensitivity under cryogenic conditions. In this manner, the inevitable problems of low concentration and poor selectivity described above can be overcome, enabling highly reliable spectroscopic identification of chemical intermediates.

**FIGURE 12:**
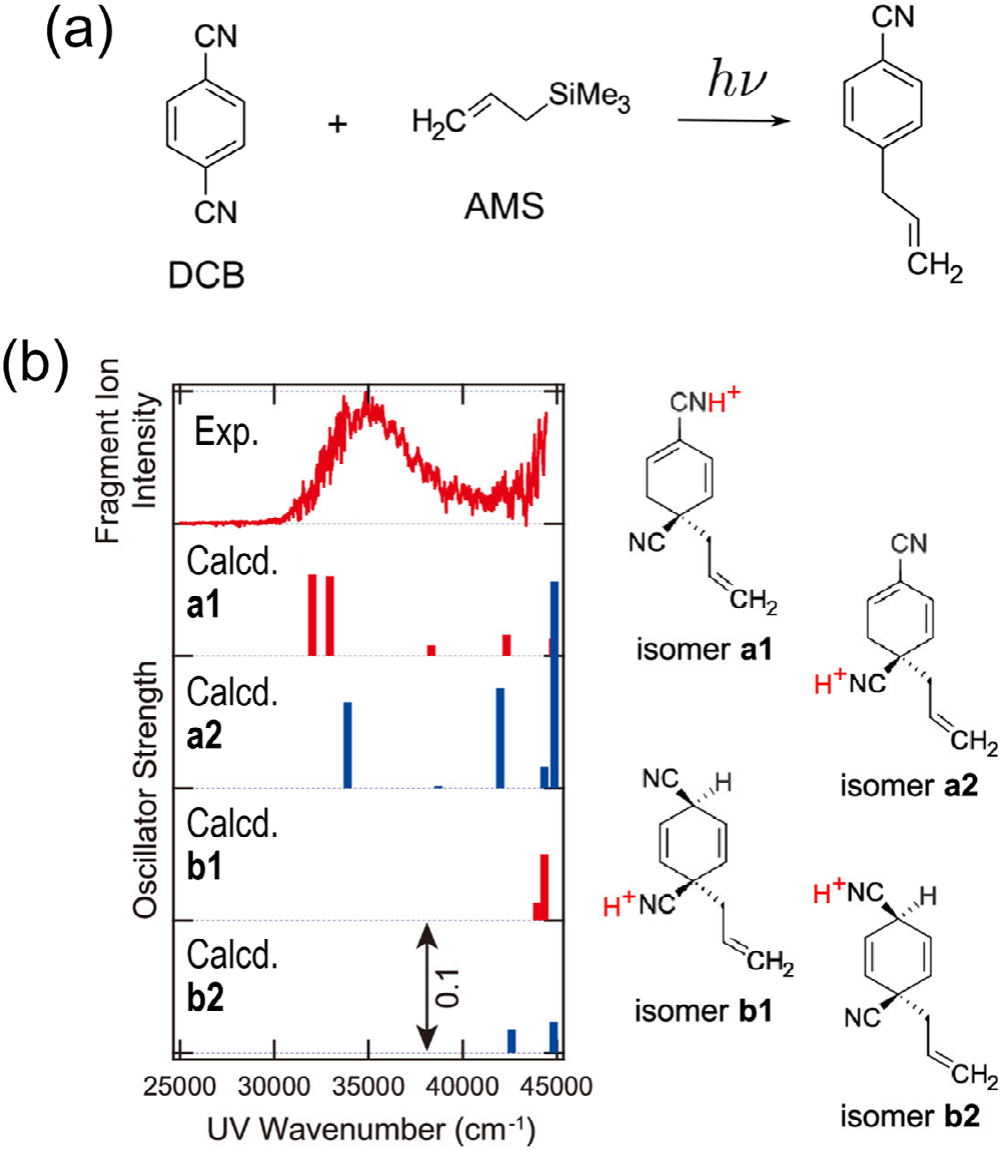
(a) Photoallylation of *p*‐DCB by AMS. (b) UVPD spectrum of chemical intermediates (C_11_H_11_N_2_^+^) and calculated electronic excitation energies (bar spectra) for structural candidates (**a1**, **a2**, **b1**, **b2**). Adapted with permission from Ref. [148].

To achieve this, an ion source designed to promptly introduce intermediates into the vacuum chamber is required. In this regard, Mayer and Asmis developed a microfluidic chip reactor that enabled the IRPD detection of intermediates in the Hantzsch cyclization [145]. Roithová et al. reported the IR spectroscopic characterization of several intermediates within catalytic cycles [146, 147]. More recently, we examined the intermediates formed during the photoallylation of *o*‐, *m*‐, and *p*‐dicyanobenzene (DCB) (Figure 12a for *p*‐DCB) using CIT‐based UVPD spectroscopy [148, 149]. This was achieved using an ESI emitter coupled with a quartz cell, following the earlier design of Arakawa et al. [150], for the prompt transfer of reaction mixtures into vacuum. The UVPD spectrum of the *m*/*z* 171 intermediate (C_11_H_11_N_2_^+^) is presented in Figure 12b. With the aid of theoretical calculations, the structure of the intermediate was assigned to **a1** and/or **a2** (Figure 12b); actually, this framework, in which the additional hydrogen is at the β position of the CN group, is best suited for the subsequent CN‐elimination step. Although *m*‐DCB is known to be much less reactive in this photoallylation, subsequent work identified a similar intermediate, even for *m*‐DCB, and revealed that the lower reactivity was attributed to a much slower step after intermediate formation [149]. As a related example, we characterized diazonium‐cation intermediates formed during the oxidation of hydrazine derivatives by developing a two‐solution mixing cell coupled with an ESI emitter and isolating the ions in a CIT for UVPD analysis [151].

## 4 Summary and Outlook

In this review, we surveyed recent progress in investigations of the intrinsic chemistry of ions using CITs, with an emphasis on electronic excitation (UV–Vis) spectroscopy. We outlined the diversity of trap geometries, action‐detection schemes, and advanced (often multi‐color) methods. Additionally, we highlighted case studies elucidating vibronic structures, conformations, bonding characteristics, and excited‐state dynamics over a wide range of ionic molecular systems, including carbocations and protonated hydrocarbons, organic dyes, host‐guest complexes, micro‐hydrated ions, hypervalent ions, metal clusters, and ionic intermediates formed in solution.

Prospectively, cryogenic gas‐phase spectroscopy will continue to provide molecularly precise information that is difficult to access in condensed media, offering a molecule‐level understanding of how and under what conditions molecular function arises in practical environments. Additionally, this powerful technique is expected to uncover “latent” molecular functions that have previously been masked by complex surroundings. Establishing CIT‐based ultra‐clean‐environment measurements as reliable reference standards for the broader chemistry community, including synthetic, catalytic, biomolecular, and material chemistry, will build closer links with these fields, where gas‐phase data provide guiding principles for rational design of functional materials. To this end, continued methodological and instrumental development will be crucial for further expanding the scope of gas‐phase chemistry.

## Conflicts of Interest

The authors declare no conflicts of interest.
